# The REVAMP natural experiment study: the impact of a play-scape installation on park visitation and park-based physical activity

**DOI:** 10.1186/s12966-017-0625-5

**Published:** 2018-01-25

**Authors:** Jenny Veitch, Jo Salmon, David Crawford, Gavin Abbott, Billie Giles-Corti, Alison Carver, Anna Timperio

**Affiliations:** 1 Institute for Physical Activity and Nutrition (IPAN), School of Exercise and Nutrition Sciences, Deakin University, Geelong, Australia; 2 RMIT University, Centre for Urban Research, Melbourne, Australia; 3 School of Exercise and Nutrition Sciences, Deakin University, Geelong, Australia; 4 Mary MacKillop Institute for Health Research, Australian Catholic University, Melbourne, Australia

**Keywords:** Natural experiment, Park refurbishment, Play-scape, Park visitation, Physical activity

## Abstract

**Background:**

Designing parks that optimise visitation and support visitors to be active is important for public health. Yet there is very little evidence about whether playground refurbishment achieves these objectives. This study examined the impact of the installation of a play-scape in a large metropolitan park in Melbourne, Australia.

**Methods:**

Natural experiment study (intervention vs control). At both parks, park visitation and physical activity were assessed before (T1, 2013) and after the intervention at 12 (T2, 2014) and 24 months (T3, 2015). At each time point, measures included: observations of park visitors using the System for Observing Play and Recreation in Communities on four weekdays and four weekend days, objective monitors to record usage of the walking paths and the number of cars entering the park; and intercept surveys with adult park visitors. Cross-sectional surveys were conducted with local residents at T1 and T3.

**Results:**

The observational data showed a 176% increase in park visitor counts from T1 to T2 (Incidence Rate Ratio (IRR) = 2.76, 95% CI = 1.04–7.33), at the intervention park relative to the control park. The intervention park had a 119% increase in counts of visitors observed engaging in MVPA from T1 to T2 (IRR = 2.19, 95% CI = 1.14–4.20), and a 128% increase from T1 to T3 (IRR = 2.28, 95% CI = 1.19–4.38), relative to the control park. The relative increases in visitation  at the intervention park play-scape compared with the control park playground were highly statistically significant from both T1 to T2 (IRR = 18.12, 95% CI = 5.51–59.59) and T1 to T3 (IRR = 15.05, 95% CI = 4.61–49.16). Similarly, there was a significant interaction between time and park with regard to the number of visitors observed engaging in MVPA in the play-scape/playground areas. The intercept survey data showed an increased odds of children’s regular visitation to the intervention park at T2 (OR = 2.67, 95% CI = 1.08, 6.64), compared with T1, relative to the control park. The remaining results from the intercept survey, objective monitors and resident surveys showed no significant differences in visitation between the two parks.

**Conclusions:**

These findings confirm that a well-designed play-scape installation has the potential to increase park visitation and encourage visitors to be physically active.

**Trial registration:**

Current controlled trial ISRCTN50745547.

**Electronic supplementary material:**

The online version of this article (10.1186/s12966-017-0625-5) contains supplementary material, which is available to authorized users.

## Background

Physical inactivity is a major contributor to the burden of chronic disease [1]. The potential of the built environment to influence physical activity is well recognised [2]. Public open spaces including parks are important settings that provide opportunities for physical activity across diverse population groups [3]. Attracting residents to visit and to be physically active in parks is therefore an important public health goal. Given significant forecasted urban population growth and increases in mid and high density living [4, 5], the availability of high quality parks is critical for future generations. Previous research has shown park proximity, size, quality and facilities to be associated with visitation and park-based physical activity across the lifespan [6, 7]. Other co-benefits such as physical health, mental health, social benefits, safety/injury prevention, and environmental sustainability have also been identified [8]. Parks receive significant financial investment from government for modifications and maintenance, and these changes are typically long-lasting. Yet there is very little evidence internationally about whether park refurbishment or renewal increases park visitation and park-based physical activity [9].

Testing the impact of park refurbishment or renewal via investigator-led experimental studies is likely to be costly and may not be feasible. Natural experiments, in contrast, involve researchers evaluating the effectiveness of ‘real world’ changes in the physical environment, social environment and/or political world that have not been influenced by the researcher [10], and are therefore significantly less costly. Natural experiments have been identified as a priority for investigating causal associations between the built environment and physical activity [11], but are conducted infrequently.

A recent review of natural experiments that evaluated the impact of environmental change in urban green space on visitation and physical activity indicated that 44% of studies (4 of 9) that examined interventions focused only on the built environment showed a positive effect for increasing park visitation and physical activity [9]. All three studies that examined combined approaches of changes in the built environment with physical activity programs showed a positive effect [9]. However, more research measuring the impact of park improvements in diverse parks and neighbourhoods is needed [9, 12]. For example, a study in the USA that examined the impact of significant renovations to playfields (mainly used for soccer and baseball) in two public parks as well as programming, and training and skill development for park and recreation program staff showed significant increases in visitation with over a 4-fold increase in the average number of visitors per observation among most age groups [13]. The evaluation of a promotion campaign of a newly constructed Rail Trail in Australia showed that mean cycling time increased among cyclists in the intervention group while mean cycling tine decreased among cyclists in the control area [14]. A study in the USA investigated the impact of a physical activity promotion to encourage use and physical activity in urban green spaces in which 51 parks were allocated $4000 to spend on park programs which included signage, promotional incentives and outreach activities. Results showed a significant increase in physical activity and number of park users, generating an estimated average of 600 more visits/week/park and 1830 more MET-hours of PA/week/park [15].

Only a small number of studies internationally have specifically examined the impact of an installation or re-development of a playground on park visitation and physical activity and to our knowledge no previous studies have examined the impact of the installation of a playground alone without significant other built environment or program changes occurring simultaneously. An Australian study examining the impact of playground renovations (as well as new greenery, lighting, park furniture and access to an adjacent sports field) among children aged 2–12 years found no detectable differences in park use or the number of children engaging in moderate- to vigorous-intensity physical activity (MVPA) nine months post-playground refurbishment between the intervention park and a control park [16]. In that study, the impact of the playground renovation on park use among visitors older than 12 years was not examined. In contrast, another Australian natural experiment examined the impact of improvements in a small neighbourhood park (including the installation of a modest playground, walking path, landscaping and a fenced dog off-leash area) and showed significant increases in park visitation (>300% increase) and the number of people engaging in vigorous park-based physical activity (>500% increase) following park refurbishment compared with a control park [17]. In the USA, a study that examined the impact of park renovation including the installation of new play equipment (as well as other features such as landscaping, ground surfaces, outdoor fitness equipment and an indoor recreation centre) found that the number of visitors more than doubled and estimated energy expenditure in the two renovated parks increased substantially compared with the control parks, with greater increases in visitation observed among children and adults than adolescents and seniors [18].

It is important to examine the impact of park refurbishment across the life course as adolescents and older adults are under-represented among park visitors [19] and it is unknown whether play equipment installation will result in increases in park use and park-based physical activity across a range of ages. It is also unclear whether such improvements will primarily attract local residents or visitors from further afield.

Further, whilst parkland appears to be equitably distributed across different suburbs in Melbourne [20], the quality of parks is not. Parks in socio-economically disadvantaged areas have fewer amenities likely to promote physical activity than parks in wealthier areas [21], therefore improving parks is likely to be advantageous for increasing the amount of physical activity among disadvantaged populations where residents are at an increased risk of inactivity and associated poor health [22]. Therefore, further research to examine the impact of improvements in parks located in low socio-economic status (SES) areas is needed.

This paper reports on the outcomes of a natural experiment involving installation of a children’s play-scape in a large metropolitan park located in a low SES area of Melbourne, Australia. Specifically, compared to a control park, we examined the impact of a play-scape installation on park visitation and park-based physical activity among children, adolescents, adults and older adults visiting the park and also among local residents living or with children attending a school, close to the park. We hypothesise that the installation of a play-scape in a park will increase visitation compared to a control park without the installation. It is also hypothesised that having increased visitors in the park with result in increased physical activity through walking and other forms of physical activity compared to the control park. This research is relevant to public health because park-based physical activity contributes to overall levels of physical activity and has additional benefits of spending time in greenspace.

## Methods

### Study background and evaluation

An opportunity for a natural experiment arose through discussions with a state-based government organisation (Parks Victoria) which manages State parks, reserves, waterways and other public land in Victoria, Australia. Parks Victoria was planning to install a play-scape (a play area designed with the intent of bringing children and accompanying adults back to nature) in a large metropolitan park.

The Recording and EValuating Activity in a Modified Park (REVAMP) study was designed to evaluate the impact of the park modification by using multiple measures to comprehensively assess park visitation and park-based physical activity in the intervention park and in a control park. More detailed information on the study methods have been provided elsewhere [12]. Measures included observational data of park visitors, objective monitoring of path usage within the parks and of vehicles entering on-site carparks, intercept surveys with adult park visitors, and two cross-sectional surveys with local residents.

Baseline assessments were conducted in April–May (Autumn) 2013 (T1), the park improvement occurred between September 2013–February 2014, first follow-up measures were conducted in April–May 2014 (T2) and second follow-up measures were conducted in April–May 2015 (T3). Each data collection took place at the same time of the year to account for potential seasonal effects.

### Study setting

The intervention park (329 ha) is located 28 km north-west of Melbourne’s central business district (CBD) in a low SES area. The control park (120 ha) is located 22 km east of Melbourne’s CBD in a high SES area and is approximately 35 km from the intervention park via the road network. It was not possible to find a matching large park in a disadvantaged area with similar features to the intervention park that was not undergoing any refurbishment during the study period. Despite the differences in overall size and SES, at baseline these two parks provided similar infrastructure and settings for being active, such as extensive walking/cycling paths, grassy open space areas and basic playground equipment. In addition, both parks had other supportive amenities to encourage visitation such as toilets, car-parking and a variety of picnic shelters, tables and barbeque areas. More details on the park features have been provided elsewhere [23].

### The park refurbishment

The refurbishment at the intervention park involved the installation of an innovative AUD$1.1 million play-scape suitable for children of all abilities that was designed by a landscape architect sourced by Parks Victoria. The new equipment included a large 360 degree swing, traditional swing set, maze, rockers, sandpit, nature play area, climbing equipment, landscaping, and various sculptures and was designed to be accessible for children with disabilities. The play-scape was also designed to encourage visitors to connect with both the natural environment and the significant indigenous cultural heritage of the region with references to local flora, fauna, past farming practices and key indigenous stories throughout the play-scape. Prior to refurbishment, the area where the play-scape was built was an open space area with no features or amenities. The playground at the control park was an older style adventure playground which included play equipment such as: slides, swings, climbing equipment, fireman’s pole, and swing bridges. See Additional file 1 for images of the new play-scape at the intervention park and the playground at the control park.

### Measures

#### Observations of park visitors

Direct observations of park visitors were conducted using a modified version of the System for Observing Play and Recreation in Communities (SOPARC) to obtain counts of the number of people in the park and the activity in which they were engaging [24]. SOPARC is a reliable, objective observation tool for assessing physical activity in community settings that is often used to specifically assess visitation and physical activity in parks [19]. It is based on momentary time sampling and involves undertaking systematic scans (an observation sweep moving from left to right) of each participant within a target area at a particular time. Prior to each time-point, observers were trained to use SOPARC in a classroom workshop and on-site parks visits. Strong inter-rater reliability was obtained following the training; 92% of scans at T1, 96% at T2 and 99% at T3 had at least 80% agreement between the observers and 86% of scans at T1, 94% at T2 and 95% at T3 had 100% agreement.

Research staff conducted observation scans of pre-determined target areas that were identified after discussions with the park rangers to determine the most highly visited areas and included, for example, the playground site, walking/cycling paths, grassy open spaces, shelters and picnic areas. There were 10 target areas in each park at T1. At T2 and T3, the area where the play-scape was installed was split into five target areas to enable more accurate observations of play-scape users. This resulted in 14 target areas at the intervention park at T2 and T3. The days and times of data collection were the same for both parks at each time-point (eight days, including four weekdays and four weekend days). During weekdays, observations were conducted every hour from 7:30 am-4:30 pm (except for one day at T1 when observations concluded at 1:30 pm due to rain), and on weekend days every hour from 8:30 am-4:30 pm. At T1 a total of 730 scans at each park (10 target areas * 73 time-points) were completed. At T2 and T3, 1064 scans (14 target areas * 76 time-points) were completed at the intervention park and 760 scans (10 target areas * 76 time-points) at the control park. This equated to a total of 5108 scans across both parks.

During each scan, research staff recorded each individual in view within their target area according to: their estimated age group (i.e. child (1–12 yrs), teen (13–20 yrs), adult (21–59 yrs), or older adult (60 yrs.+)); sex (male or female); and the activity they were engaged in (lying down or sitting, standing, moderate activity (e.g. walking), or vigorous activity (e.g. jogging, cycling)).

Observations were not conducted on days of forecasted rain; however, unexpected rain showers occurred on some days. The variation in average hourly temperature and rainfall during the observation periods (i.e. 7.30 am-4.30 pm on weekdays) between the intervention and control parks and across the three time-points was minimal (data obtained from the Bureau of Meteorology). The average hourly temperature and rainfall during the observation periods at the intervention and control parks are presented in Additional file 2.

#### Electronic path monitors and car traffic counters

Both parks contained a network of sealed paths. Electronic path monitors were used to record counts of people walking and cycling on two pre-selected paths on the same days observations were conducted. The monitors were positioned at areas that were used most frequently and were most comparable between the two parks. Further details are provided elsewhere [12]. The path monitors were set up at 7:30 am on weekdays and 8:30 am on weekend days and were removed at 4:30 pm each day. Total counts for the two monitors at each park were calculated for the eight days of data collection at each time-point.

There was one main entrance to each park. At both parks a traffic counter was located at this entrance to record the number of vehicles entering and exiting the parks (hourly counts) on the days when park observations were conducted. Total counts of traffic entering each park from 7 am-5 pm were calculated for the eight days of data collection at each time-point.

#### Intercept surveys

Face-to-face intercept interviews were completed with English-speaking adult park visitors on days when observations were conducted (for logistic reasons it was not possible to have translators available in the park). The intercept surveys provided an opportunity to gain more detailed information about the park visit than could be obtained from the observations. Trained, clearly identifiable research assistants approached park users in the specified target areas, explained the study and all ethical considerations, and invited participation. At T1, 794 park visitors completed an interview (75.3% of those approached, excluding 201 park visitors who had already been intercepted); at T2,  1158 park visitors completed an interview (71.2% of those approached, excluding 293 park visitors who had already been intercepted at T2); and at T3,  1043 park visitors completed an interview (74.3% of those approached, excluding 371 park visitors who had already been intercepted at T3).

Park visitors were asked their age and sex and how often they had visited the intervention/control park in the past three months (daily, 2–3 times/week, once/week, 2–3 times/month, once/month, <once/month, have not visited in past three months). For the purpose of analysis these response options were collapsed to: ≥once/week; once/month to 2–3 times/month; or <once/month. They also reported their usual activity levels during visits to the park in the past three months (mostly sitting, mostly light activities, mostly moderate activities, or mostly vigorous activities).

Participants were also asked if they had a child(ren) aged 2–15 years, and if so, they were asked to consider the child in the age range who had the next birthday and report that child’s age and sex and how often that child had visited the intervention/control park in the past three months. Response options were the same as those described above for adults.

#### Resident surveys

Cross-sectional surveys were completed by adult residents at T1 and T3. These surveys provided a population estimate of park visitation rather than relying solely on observation or park intercepts, which only captures visitors and may also capture repeat visitors. Recruitment was via two methods: 1) families with children attending pre-schools, primary and secondary schools located within 3 km of each park; and 2) a postal survey from the local City Council to households located within 5 km of each park. At T1, 9694  surveys were delivered, 37 were returned to sender (no longer resided at that address) and removed from the denominator, and 1487 surveys were returned completed (15.4% response rate; 15.1% intervention park, 15.7% control park). At T3, 9537  surveys were delivered,  44 were returned to sender and removed from the denominator, and  1460 were returned completed (15.4% response rate; 14.1% intervention park, 16.6% control park).

The survey included socio-demographic variables (age, sex, country of birth, number of children, highest level of education, employment status, marital status, and dog ownership). Participants also reported how often they had visited the intervention/control park in the past three months (daily, 2–3 times/week, once/week, 2–3 times/month, once/month, <once/month, had not visited in past three months). These response options were collapsed to: ≥once/week; once/month to 2–3 times/month; or <once/month. They also reported how long they were usually active on each park visit in the past three months (minutes) and their usual activity levels during visits to the park in the past three months (mostly sitting, mostly light activities, mostly moderate activities, or mostly vigorous activities). Time spent (minutes) in transportation and leisure-time physical activity in the last seven days was examined using the long form of the International Physical Activity Questionnaire (IPAQ-L) [25].

Respondents with a child(ren) aged 2–15 years living in the household, were asked to complete proxy-report survey questions on behalf of their child (next birthday method) regarding age and sex, how often their child had visited the intervention/control park in the past three months, how long their child was usually active for on each park visit in the past three months, and their child’s usual activity levels during visits to the park in the past three months. Response options were the same as those described above for adults.

### Statistical analysis

Descriptive statistics for overall observation visitor counts, observation visitor counts in the new play-scape at the intervention park and playground at the control park, path monitor counts, traffic counts, intercept surveys and resident surveys for the two parks at each time-point were calculated.

Analyses of the observation and traffic data included three separate count outcomes with hourly counts as the unit of analysis; overall number of visitors observed, number of people observed in MVPA and traffic counts. There were insufficient cases to run inferential analyses for the path monitor data as path monitor counts were recorded as total daily counts. For each of these three outcomes, a multilevel negative binomial regression model was conducted with random intercepts for measurement days (i.e. accounting for clustering of hourly observations within measurement days at each park). Models included main effects of time (T1/T2/T3) and park (intervention/control), as well as a time by park interaction. The time by park interaction was used to assess the effect of park refurbishment. As the time factor had three values, two interaction coefficients were produced for each model. T1 (2013) was set as the reference value for time and the control park set as the reference park. These two coefficients represented differences in outcomes at the intervention park; firstly between T1 and T2, and secondly between T1 and T3, relative to the control park. As the outcome variables were counts, the interaction effects have been reported as Incidence Rate Ratios (IRRs). The models adjusted for the following covariates: hourly temperature; hourly rainfall; and whether it was a weekday or weekend day. All analyses were conducted using Stata/SE 14 (StataCorp, TX).

Logistic regression models were used to test the effect of the park refurbishment on odds of regular visitation (>once/week over the past three months) among adult participants (and their children) who completed the intercept surveys and, separately, among adult participants (and their children) who completed the resident surveys and had visited the intervention/control park in the past three months. Models included main effects for park and time-point, their interaction, and potential confounders of age and sex. Statistical significance of the interaction term was used to determine if the outcome varied between the two parks at T2 (intercept surveys only) or T3 relative to their baseline difference. This method is also known as difference-in-difference analysis [3]. Equivalent logistic regression models were also used to examine effects of the park refurbishment on odds of adult participants (and their children) engaging primarily in MVPA while in the park, among those who reported (on behalf of themselves and their child) that they had visited the intervention/control park in the past three months. Finally, among those who had visited the park in the past three months, linear regression models (with main effects for park and time-point as well as their interaction) were used to examine effects of the park refurbishment on the time in minutes adult respondents (on behalf of themselves and their child) reported they were usually active on each park visit.

## Results

### Observations of park visitors

Table 1 shows overall park visitor counts and counts stratified by sex, age group, weekday/weekend days, and activity levels for the intervention and control parks at the three time-points. Total visitor counts at the intervention park increased by 33% from T1 to T2 then remained stable to T3; an 11% decline in visitor counts was observed at the control park from T1 to T2, and a 22% decline from T2 to T3. Similar numbers of males and females were observed at all time-points. The percentage of children (1–12 years) observed increased by 60% at the intervention park from T1 to T3, and the percentage of older adults (60+ years) observed decreased from T1 to T3 at both parks. More people were observed on weekend days than weekdays. The percentage observed engaged in MVPA remained relatively stable at the intervention park from T1 to T2 but decreased at the control park from T1 to T3.

**Table 1:** Counts of park visitors observed at the intervention and control parks at the three time-points

	Intervention Park			Control Park		
	T1 n (%)	T2 n (%)	T3 n (%)	T1 n (%)	T2 n (%)	T3 n (%)
Total visitor counts	2374	3162	3157	2382	2130	1654
Average hourly counts (Mean ± Std Err*)*	32.5 ± 5.1	41.6 ± 6.3	41.5 ± 6.4	32.6 ± 3.9	28.0 ± 4.8	21.8 ± 2.9
Sex						
Female	1177 (49.6)	1618 (51.2)	1639 (51.9)	1263 (53.0)	999 (46.9)	852 (51.5)
Male	1197 (50.4)	1544 (48.8)	1518 (48.1)	1119 (47.0)	1131 (53.1)	802 (48.5)
Age (years)						
Child (1–12)	434 (18.3)	960 (30.4)	922 (29.2)	678 (28.5)	616 (28.9)	609 (36.8)
Teen (13–20)	188 (7.9)	163 (5.2)	222 (7.0)	165 (6.9)	84 (3.9)	77 (4.6)
Adult (21–59)	1325 (55.8)	1604 (50.7)	1674 (53.0)	1217 (51.1)	1211 (56.9)	877 (53.0)
Older adult (60+)	427 (17.9)	435 (13.8)	339 (10.7)	322 (13.5)	219 (10.3)	91 (5.5)
Day of week						
Weekdays	256 (10.8)	319 (10.1)	332 (10.5)	563 (23.6)	234 (11.0)	482 (29.1)
Weekend days	2118 (89.2)	2843 (89.9)	2825 (89.5)	1819 (76.4)	1896 (89.0)	1172 (70.9)
Activity levels						
Lying down/sitting	615 (25.9)	872 (27.6)	1051 (33.3)	553 (23.2)	588 (27.6)	491 (29.7)
Standing	970 (40.8)	1233 (39.0)	1199 (38.0)	801 (33.6)	800 (37.6)	580 (35.1)
Moderate	633 (26.7)	882 (27.9)	785 (24.9)	738 (30.9)	570 (26.8)	437 (26.4)
Vigorous	156 (6.6)	175 (5.5)	122 (3.9)	290 (12.2)	172 (8.1)	146 (8.8)
MVPA	789 (33.2)	1057 (33.4)	907 (28.7)	1028 (43.2)	742 (34.8)	583 (35.2)

The number of hourly observation windows was 76 at T2 and T3, but only 73 at T1 due to missing data

*MVPA* moderate- vigorous-intensity physical activity

**Fig. 1:**
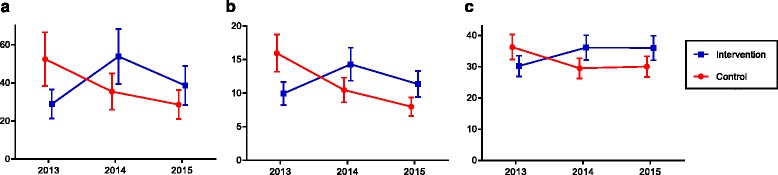
Marginal means of total observations, people observed in moderate- and vigorous-intensity physical activity and number of cars entering parks, by park and time-point. Error bars represent standard error of the mean. **a**. Number of people per hour. **b**. Num ber of people being active per hour. **c**. Number of cars entering park per hour

There was a significant interaction between time and park with regard to total park visitors. The intervention park had a 176% increase in counts of park visitors from T1 to T2 (IRR = 2.76, 95% CI = 1.04–7.33, *p* = 0.042), relative to the control park (see Fig. 1a). That is, counts of park visitors from T1 to T2 increased by 176% more in the intervention park compared to the control park. The interaction effect for differences in counts of park visitors between T1 and T3, however, did not reach statistical significance (IRR = 2.45, 95% CI = 0.92–6.50, *p* = 0.071). When these analyses were conducted separately for different age groups, relative to the control park, the total number of children (IRR = 6.10, 95% CI = 1.91–19.48, *p* = 0.002) and adult visitors (IRR = 2.23, 95% CI = 1.16–4.29, *p* = 0.016) increased significantly more in the intervention park from T1 to T2. There were also significant increases in the total number of children (IRR = 4.54, 95% CI = 1.42–14.54, *p* = 0.011) and adult (IRR = 2.11, 95% CI = 1.10–4.06, *p* = 0.025) visitors observed at the intervention park from T1 to T3, relative to the control park. No significant time by park interactions were found in the number of adolescents and older adult visitors observed.

There was a significant interaction between time and park with regard to park visitors observed engaging in MVPA. The intervention park had a 119% increase from T1 to T2 (IRR = 2.19, 95% CI = 1.14–4.20, *p* = 0.019), and a 128% increase from T1 to T3 (IRR = 2.28, 95% CI = 1.19–4.38, *p* = 0.013), relative to the control park (see Fig. 1b). When these analyses were conducted separately for different age groups there were significant increases from T1 to T2 in engagement in MVPA at the intervention park, relative to the control park, among children (IRR = 5.15, 95% CI = 1.87–14.22, *p* = 0.002) and adults (IRR = 1.81, 95% CI = 1.06, 3.11, *p* = 0.030). There were also increases in the number of children observed engaging in MVPA at the intervention park from T1 to T3, relative to the control park (IRR = 5.44, 95% CI = 1.94–15.28, *p* = 0.001).

### Observations in the play-scape area

Visitor counts specifically for the new play-scape area in the intervention park increased substantially (more than 600%) from T1 to T3 (Table 2). As anticipated, there was a substantial increase in the percentage of play-scape users who were children from T1 (11.4%) to T3 (43.4%), with a smaller increase in adults from T1 (28.0%) to T3 (42.1%). As might be expected, the increase in adolescent users was considerably smaller from T1 (0%) to T3 (5.2%). In contrast, visitor counts at the control park’s playground decreased by approximately 13% from T1 (*n* = 448) to T2 (*n* = 460) to T3 (*n* = 390). Statistical tests of the interaction between time and park showed the relative increases at the intervention park play-scape compared with the control park playground were highly statistically significant from both T1 to T2 (IRR = 18.12, 95% CI = 5.51–59.59, *p* < 0.0005) and T1 to T3 (IRR = 15.05, 95% CI = 4.61–49.16, p < 0.0005). Similarly, there was a significant interaction between time and park with regard to the number of visitors observed engaging in MVPA in the play-scape/playground areas. The relative increase at the intervention park play-scape compared with the control park playground were highly significant from both T1 to T2 (IRR = 14.45, 95% CI = 4.15–50.28, p < 0.0005) and T1 to T3 (IRR = 24.19, 95% CI = 6.79–86.19, p < 0.0005).

**Table 2:** Counts of park visitors observed in the play-scape area at the intervention park and playground at the control park at the three time-points

	Intervention Park			Control Park		
	T1 n (%)	T2 n (%)	T3 n (%)	T1 n (%)	T2 n (%)	T3 n (%)
Total visitor counts	132	1112	1016	448	460	390
Average hourly counts (Mean ± Std Err*)*	1.8 ± 0.4	14.6 ± 2.3	13.4 ± 2.2	6.1 ± 0.9	6.1 ± 1.0	5.1 ± 0.7
Sex						
Female	74 (56.1)	591 (53.1)	569 (56.0)	232 (51.8)	237 (51.5)	197 (50.5)
Male	58 (43.9)	521 (46.9)	447 (44.0)	216 (48.2)	223 (48.5)	193 (49.5)
Age (years)						
Child (1–12)	15 (11.4)	524 (47.1)	441 (43.4)	275 (61.4)	291 (63.3)	245 (62.8)
Teen (13–20)	0 (0)	45 (4.0)	53 (5.2)	11 (2.5)	17 (3.7)	10 (2.6)
Adult (21–59)	37 (28.0)	426 (38.3)	428 (42.1)	116 (25.9)	143 (31.1)	134 (34.4)
Older adult (60+)	80 (60.6)	117 (10.5)	94 (9.3)	46 (10.3)	9 (2.0)	1 (0.3)
Day of week						
Weekdays	5 (3.8)	160 (14.4)	181 (17.8)	66 (14.7)	76 (16.5)	72 (18.5)
Weekend days	127 (96.2)	952 (85.6)	835 (82.2)	382 (85.3)	384 (83.5)	318 (81.5)
Activity levels						
Lying down/sitting	11 (8.3)	280 (25.2)	318 (31.3)	24 (5.4)	52 (11.3)	60 (15.4)
Standing	42 (31.8)	439 (39.5)	361 (35.5)	90 (20.1)	181 (39.3)	172 (44.1)
Moderate	76 (57.6)	343 (30.8)	287 (28.2)	247 (55.1)	166 (36.1)	95 (24.4)
Vigorous	3 (2.3)	50 (4.5)	50 (4.9)	87 (19.4)	61 (13.3)	63 (16.2)
MVPA	79 (59.8)	393 (35.3)	337 (33.2)	334 (74.5)	227 (49.3)	158 (40.5)

The number of hourly observations windows was 76 at T2 and T3, but only 73 at T1 due to missing data

*MVPA* moderate- vigorous-intensity physical activity

### Electronic path monitors and car traffic counters

Path monitor counts increased from T1 to T2 at the intervention park (Table 3). As noted previously, there were insufficient cases to run inferential analyses for the path monitor data as path monitor counts were recorded as total daily counts. Traffic counts increased over the three time-points at the intervention park with a decline in traffic counts observed at the control park; however, the differences in counts at each time-point at the intervention park relative to the control park were not statistically significant (see Fig. 1c).

**Table 3:** Total path monitor and traffic counts at the intervention and control parks at the three time-points

	Intervention Park			Control Park		
	T1	T2	T3	T1	T2	T3
Path monitor counts	1137	1499	1495	6067	6030	6541
Traffic counts	2336	2780	2903	2995	2325	2439

### Intercept surveys with park visitors

Descriptive results for the intercept surveys are presented in Table 4. Among adults, self-reported regular visitation (≥once/week over the past three months) was approximately 18% lower at the intervention park at T3 than at T1, but did not vary over the three time-points at the control park (T1: 37.4%; T2: 34.7%; T3: 36.5%). However, the differences in self-reported visitation at each time point between the intervention and control parks were not statistically significant. Proxy-reported regular visitation (≥once/week over the past three months) by children was 52% higher at the intervention park at T3 compared with T1, whereas at the control park proxy-reported regular visitation by children was 20% lower at T3 compared with T1. There was a significant park by time interaction, with increased odds of children’s regular visitation to the intervention park at T2 (OR = 2.67, 95% CI: 1.08, 6.64, *p* = .034), compared with T1, relative to the control park. The effect for T3 versus T1 did not reach significance (OR = 2.31, 95% CI: 0.90, 5.96, *p* = .082).

**Table 4:** Park visitation at the intervention and control parks from the park-based intercept surveys at the three time-points

	Intervention Park			Control Park		
	T1 (*n* = 313)	T2 (*n* = 597)	T3 (*n* = 485)	T1 (*n* = 481)	T2 (*n* = 561)	T3 (*n* = 558)
Age years (mean (SD))	46.2 (15.0)	45.0 (14.6)	44.4 (15.3)	46.4 (14.4)	46.1 (15.2)	46.4 (15.4)
Child’s age (mean (SD))	7.5 (4.0)	6.7 (3.9)	6.9 (3.9)	6.6 (3.7)	6.8 (4.1)	6.4 (3.8)
Sex (%)						
Female	48.7	47.2	50.4	61.1	52.4	59.0
Male	51.3	52.8	49.6	38.9	47.6	41.0
Sex of child (%)						
Female	36.1	50.2	46.3	53.6	52.6	43.6
Male	63.9	49.8	53.7	46.4	47.4	56.4
Adult’s usual park visitation in past 3 months (%)						
≥ once per week	36.2	34.5	29.7	37.4	34.7	36.5
Once p/month to 2–3 times p/month	23.4	23.0	26.4	17.8	23.2	21.5
< once per month	40.4	42.5	44.0	44.9	42.2	42.1
Child’s usual park visitation in past 3 months (%)						
≥ once per week	8.6	15.6	13.1	20.6	16.5	16.4
Once p/month to 2–3 times p/month	35.6	28.1	26.1	36.1	33.5	26.9
< once per month	55.8	56.3	60.8	43.3	50.0	56.6
Adult’s usual activity level during park visits in past 3 months (%)						
Mostly sitting	5.2	3.3	3.5	9.3	2.1	2.5
Mostly light activities	44.2	34.9	39.2	33.6	30.5	23.8
Mostly moderate activities	46.3	52.9	46.9	48.3	51.2	56.5
Mostly vigorous activities	4.3	8.9	10.5	8.8	16.3	17.3

A lower percentage of adults reported usually engaging in MVPA at both the intervention and control parks at T1 compared with T2 and T3. There was a significant park by time interaction, with intervention park users having reduced odds of engaging in MVPA at T2 (OR = 0.63, 95% CI: 0.41, 0.98, *p* = .039) and T3 (OR = 0.39, 95% CI: 0.25, 0.61, *p* < .0005), compared with T1, relative to the control park.

### Resident surveys

Among adult residents living within 5 km of the parks, 46.4% and 39.6% reported visiting the intervention park in the past three months at T1 and T3 respectively, and 47.8% and 41.8% had visited the control park respectively. Parents reported that 46.6% and 44.2% of children had visited the intervention park in the past three months at T1 and T3 respectively, and 47.8% and 46.9% of children had visited the control park respectively. Descriptive results for the resident surveys for those who had visited the intervention/control park in the past three months are presented in Table 5 (adults) and Table 6 (children).

**Table 5:** Adult park visitation at the intervention and control parks from the resident surveys at T1 and T3 among adults who had visited the park in the past 3 months

	Intervention Park		Control Park	
	T1 (*n* = 294)	T3 (*n* = 256)	T1 (*n* = 374)	T3 (*n* = 318)
Age years (mean (SD))	48.4 (12.7)	48.5 (13.0)	49.0 (13.4)	49.8 (13.3)
Sex (%)				
Female	64.7	69.1	71.2	69.7
Male	35.3	40.0	28.8	30.3
Country of birth (%)				
Born in Australia	66.1	62.2	72.8	62.7
Born elsewhere	33.9	37.8	27.2	37.3
Children ≤ 15 years (%)	65.4	59.1	71.5	65.8
Education level (%)				
No formal qualifications	14.6	14.2	5.2	7.4
Year12/apprentice/diploma	34.0	35.2	25.7	20.3
University degree/ higher degree	51.4	50.6	69.1	72.3
Employment status (%)				
Working full time	36.8	38.9	33.6	31.2
Working part-time	27.8	19.7	29.2	29.8
Unemployed	22.9	23.9	23.9	20.7
Retired	12.5	17.5	13.3	18.4
Marital status (%)				
Married/de-facto	83.7	81.9	85.7	84.9
Separated/widowed/divorced	11.4	11.7	10.5	13.6
Never married	4.8	6.4	3.8	1.6
Dog ownership (%)	42.9	40.2	34.5	27.1
Usual park visitation in past 3 months (%)				
≥ once per week	16.7	19.1	22.3	25.8
Once p/month to 2–3 times p/month	43.2	41.8	46.0	42.5
< once per month	40.1	39.1	31.7	31.8
How long usually active on each park visit (minutes), (mean, (SD))	58.8 (38.1)	54.8 (43.3)	52.8 (31.6)	55.1 (34.5)
Usual activity level during park visits in past 3 months (%)				
Mostly sitting	3.5	8.9	3.0	1.6
Mostly light activities	47.1	50.2	39.0	36.5
Mostly moderate activities	41.9	36.7	50.6	52.1
Mostly vigorous activities	7.6	4.2	7.4	9.8
Minutes/week of leisure-time physical activity (mean, (SD))	187.1 (311.2)	194.2 (314.3)	234.7 (299.1)	185.4 (255.9)
Minutes/week of transport physical activity (mean, (SD))	143.7 (208.8)	154.9 (234.7)	142.1 (207.9)	138.3 (213.7)

**Table 6:** Proxy-reported child park visitation at the intervention and control parks from the resident surveys at T1 and T3 among children who had visited the  park in the past 3 months

	Intervention Park		Control Park	
	T1 (*n* = 180)	T3 (*n* = 144)	T1 (*n* = 228)	T3 (*n* = 191)
Age years (mean (SD))	8.4 (3.8)	7.9 (3.3)	8.3 (3.5)	7.9 (3.3)
Sex (%)				
Female	50.0	48.2	42.6	47.3
Male	50.0	51.9	57.4	52.7
Child’s usual park visitation in past 3 months (%)				
≥ once per week	10.6	6.9	10.1	11.5
Once per month to 2–3 times p/month	43.9	44.4	54.4	50.8
< once per month	45.6	48.6	35.5	37.7
How long child usually active on each park visit (minutes), (mean, (SD))	64.7 (38.3)	71.1 (46.4)	65.1 (36.3)	66.2 (41.9)
Child’s usual activity level during park visits in past 3 months (%)				
Mostly sitting	0.6	0.0	0.0	1.1
Mostly light activities	12.4	12.9	12.5	8.8
Mostly moderate activities	80.8	76.1	82.1	81.8
Mostly vigorous activities	6.2	11.2	5.4	8.3

A lower percentage of adults reported regular visitation (≥once/week over the past three months) at T1 compared with T3 at both the intervention park and the control park. The park by time interaction effect was non-significant, suggesting no effect of park refurbishment on changes in regular visitation by local adult residents. Proxy-reported regular visitation of children was higher at the intervention park at T1 than at T3 and at the control park a slightly lower percentage of regular visitation was proxy-reported at T1 than at T3; however, again the park by time interaction effect was non-significant suggesting no effect of park refurbishment on changes in regular visitation by local child residents.

A higher percentage of adults reported usually engaging in MVPA at the intervention park at T1 than at T3, whereas at the control park a slightly lower percentage reported engaging in these activity levels at T1 than at T3. There was a significant park by time interaction (OR = 0.60, 95% CI: 0.38, 0.97, *p* = .036) suggesting a reduced odds of engaging in MVPA from T1 to T3, in the intervention park relative to the control park. Proxy-reported MVPA in children was similar at T1 and T3 at both the intervention and control park; the park by time interaction effect for children was non-significant.

There were no significant effects of park refurbishment on the duration that adult local residents reported being active on each park visit. There were also no significant effects of park refurbishment on the duration that local adults proxy-reported the time their child was usually active on each park visit (data not shown). Interestingly, there was no park by time interaction with regards to self-reported overall leisure-time or transport related physical activity among adults who had visited the intervention or control parks in the last three months.

## Discussion

This natural experiment study is one of only a few internationally that has examined the impact of the installation of a play-scape on overall park visitation and the number of park visitors and nearby residents engaged in park-based physical activity over a two-year period. Each source of rich data contributed a unique angle to better understanding the impact of park refurbishment in a low SES park and ensured a comprehensive examination of park visitation and park-based physical activity among various user sub-groups. Overall, the findings confirm that a well-designed play-scape installation has the potential to increase park visitation and encourage visitors to be physically active, but the different sources of data provided mixed findings. These findings are important for public health, as they demonstrate to those responsible for developing or redeveloping parks that the inclusion of park infrastructure such as those in this natural experiment do lead to increased activity levels by park users. Further, based on existing research, there is evidence that increased physical activity in parks will likely lead to increased overall activity.

The findings from the observation data showed a significant increase in overall park visitation in the intervention park, relative to the control park, with the changes generally persisting 12 months after the completion of the refurbishment. These results were mostly driven by the dramatic increase (670%) in visitation in the new play-scape area following the refurbishment, mainly by children and their parents. These results support previous recent natural experiment studies that showed increases in park visitation after overall park refurbishment which included playground refurbishment conducted in a variety of neighbourhood parks in Australia [17] and the United States [26]. Our findings are also consistent with a natural experiment conducted in the USA that showed greater increases in park visitation after refurbishment of a child’s play area among children and adults than in adolescents or seniors [18]. Importantly, there was also a 128% increase in park visitors observed engaging in MVPA at the intervention park, relative to the control park. These findings provide evidence that well-designed play-scape refurbishments can encourage visitation, and importantly for public health, encourage visitors to be physically active. However, results arising from other data collected as part of the evaluation varied. There was no evidence of significant effects on the number of cars entering the park; however, it is possible that park visitors entered the park using active transport via the main entrances and/or entered the park via other smaller (non-car) entrances that were available at both parks. Results from the intercept and local resident surveys showed no significant increases in regular visitation at the intervention park relative to the control park, with one exception. A significant increase in regular visitation by children at the intervention park from T1 to T2, relative to the control park, was reported in the intercept surveys. Further, the intercept survey data showed fewer adults engaging in MVPA at both T2 and T3 compared with T1, at the intervention park relative to the control park, and the resident survey results showed fewer adults engaging in MVPA from T1 to T3, at the intervention park relative to the control park.

The variation in results from the different methods highlights the importance of including multiple measures and data from different sub-groups. Previous studies of park refurbishments incorporating multiple measures have also showed inconsistent results from different measures [18]. As the observation data of park visitation is the most robust/comprehensive, it is not surprising that this measure showed significant changes in the anticipated direction. The significant differences in changes in park visitation and park-based physical activity seen in the observation data but not from residents’ survey suggests that increases in observed park visitations were not due to increased use by local residents. Rather, significant park upgrades to large metropolitan parks, may attract families and children from other areas of the city. However, response rates for both resident surveys was low (15%) and the sample is unlikely to be representative. It is plausible that there is a potential bias towards those who visit parks more regularly. Further, the placement of the path monitors in more heavily used areas (as advised by park rangers), may have reduced the ability to show increases between the various time points, due to these being in the busiest areas in the parks.

The new play-scape was designed specifically for younger children, hence, as anticipated, it was mainly used by children aged 1–12 years. There was also an increase in the number of adults in the play-scape area, most likely because of their role in supervising the children. Conversely, very few visitors in both the intervention or control parks play areas were adolescents or older adults, and there was a decline in the number of older adults observed in the play-scape from T1 to T3. Both groups are known to be under-represented among park visitors [19]. The extent to which it is possible to design parks to optimise visitation among adolescents and older adults requires further investigation [27]. For example, it is not known whether installation of play equipment designed for older youth (i.e. basketball courts, larger slides/swings, climbing equipment) would have increased visitation among this important age group [28] or what modifications would attract older adults to visit.

Further, the quality of parks located in low SES areas of Melbourne is considerably poorer than parks located in high SES areas [21], therefore the need for park upgrades in lower SES areas is warranted. This study provides evidence of the potential to reduce inequities of park quality in disadvantaged areas if those upgrades are done well.

### Strengths and limitations

Although relatively rare in public health research [9, 16, 29, 30], natural experiments are considered a priority for investigating associations between the built environment and physical activity [10]. Conducting natural experiments requires specialist expertise and knowledge and often presents conceptual and methodological obstacles [31]. Challenges experienced during the REVAMP study are discussed in more detail elsewhere [32].

While novel and unique, it is important to acknowledge that the study fındings are from one large intervention park and one large control park located in Melbourne, Australia which limits the ability to generalise the results to other parks. In addition, although the control park had similar features to the intervention park at baseline and both parks were very large parks, the control park was about one- third of the size and was located in a higher SES area than the intervention park. Given that the objective was to compare differences in changes in park visitation and park-based physical activity between the intervention and control park, the study design somewhat alleviated differences in size and SES. However, it is recognised that it is often difficult to identify a control condition that is perfectly matched to the intervention [33].

The observational data was comprehensive with observations being conducted on eight days, which is beyond the recommended minimum of four days required to obtain robust measures of park visitation [34]. The reliability data collected showed high inter-observer agreement, and there was consistency in the measures with observations completed at the same time and day at both parks in the one season. It is also important to acknowledge that direct observations are a snapshot in time that provide a general indication of park visitation on specified days and it is possible that observations conducted on other days, times and seasons may have provided different results. Further, in the current study data were only collected on fine weather days therefore it was not possible to determine if the play-scape made visitors more resilient to wet weather [35]. Previous studies of park refurbishments have used multiple measures such as observations and intercept and/or resident interviews [16, 18, 36] although to our knowledge no studies have used such a comprehensive range of measures and incorporated objective measures.

Finally, it was not possible to determine whether park use increased among original park visitors, or whether new visitors and residents from other neighbourhoods were attracted to visit the refurbished park. It was also not possible to determine whether the overall physical activity levels of park visitors actually increased or whether the park-based activity displaced activity that was previously undertaken at an alternative setting. This is important to examine in future studies. A previous review of natural experiment studies in parks described promising evidence for those combining environmental change with physical activity programs in the park [9]. Future studies may wish to examine if visitation and/or park-based physical activity could be further enhanced if the intervention was combined with activity programs held in the park.

## Conclusion

This natural experiment provides much-needed evidence that the installation of a play-scape has the potential to positively influence park visitation and park-based physical activity among children and adults attending a park in a low SES area. Increases in observed visitation at the intervention park can be largely explained by an increase in visitors to the new play-scape, which was mainly used by children aged 1–12 years. It is possible that the upgrade of a large metropolitan park attracts visitors from further away, since little change was observed in the resident survey. Understanding where the observed park users come from and how much accessing the park enhanced their activity levels is important for determining the public health outcomes of future park upgrades. The findings provide encouraging evidence for urban planners and designers that well-designed play-scape refurbishments have the potential to attract visitors and to facilitate greater levels of physical activity in the age groups for which the upgrade was targeted. However, more research is needed in parks of varying size, amenity and location and designed to attract different user groups including adolescents and older adults. Parks are important community settings, and this study provides preliminary evidence highlighting their potential for enhancing the physical health and wellbeing of residents through investment in park refurbishment.

## Additional files

Additional file 1: The intervention park prior to the play-scape installation, The new play-scape at the intervention park, Playground at the control park. (PDF 373 kb)

Additional file 2: Average hourly temperature and rainfall during the observation period at the intervention and control parks at the three time-points. (DOCX 79 kb)
