# Supplementary material for: The REVAMP natural experiment study: the impact of a play-scape installation on park visitation and park-based physical activity
Source: Int J Behav Nutr Phys Act. 2018 Jan 25;15:10. doi: 10.1186/s12966-017-0625-5 (PMC5784649; doi:10.1186/s12966-017-0625-5)

Additional File 1:

The intervention park prior to the play-scape installation

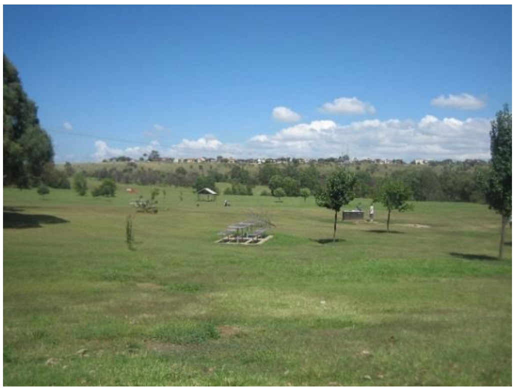

The new play-scape at the intervention park

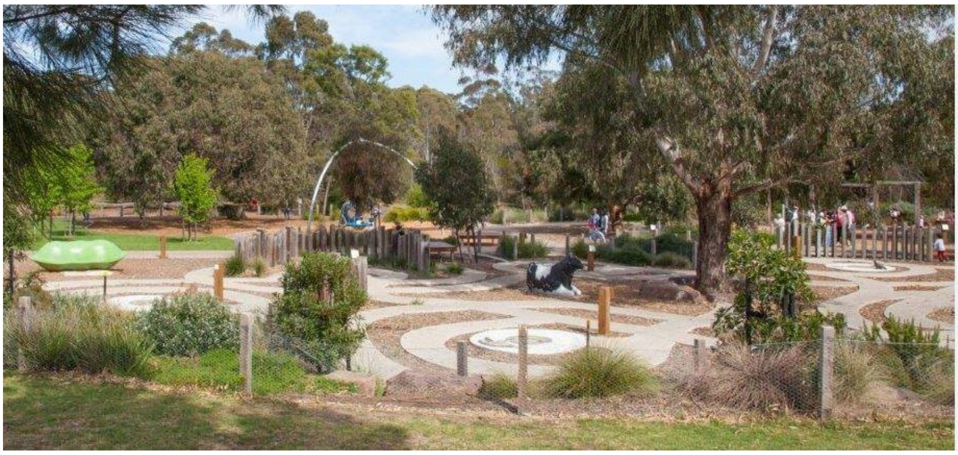

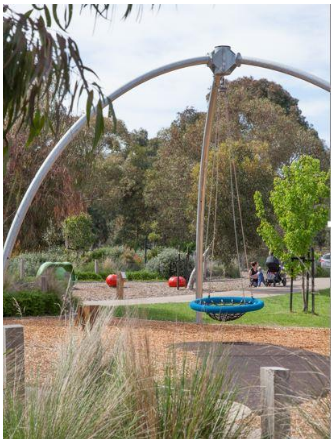

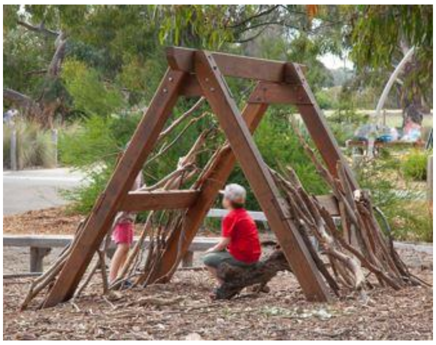

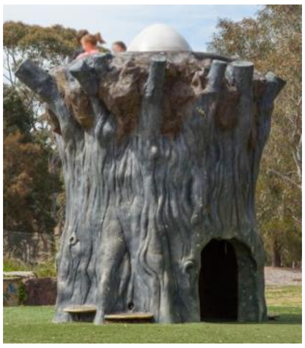

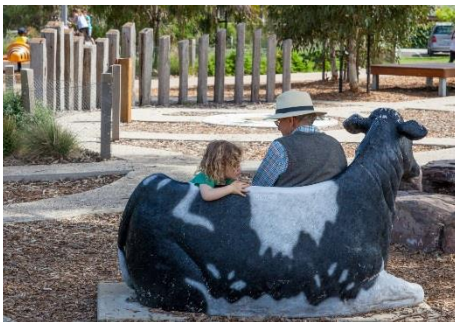

Playground at the control park

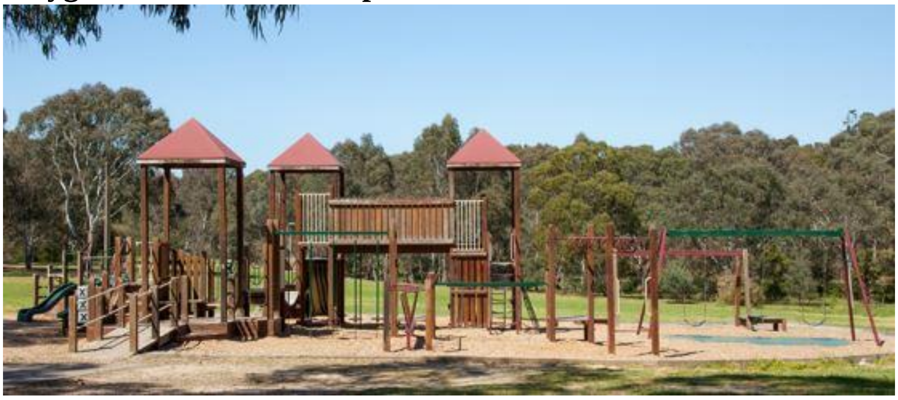

Supplement: Supplementary file 1 — The intervention park prior to the play-scape installation, The new play-scape at the intervention park, Playground at the control park. (PDF 373 kb) [file 12966_2017_625_MOESM1_ESM.pdf]
