# Supplementary material for: The REVAMP natural experiment study: the impact of a play-scape installation on park visitation and park-based physical activity
Source: Int J Behav Nutr Phys Act. 2018 Jan 25;15:10. doi: 10.1186/s12966-017-0625-5 (PMC5784649; doi:10.1186/s12966-017-0625-5)
Supplement: Supplementary file 2 [file 12966_2017_625_MOESM2_ESM.docx]

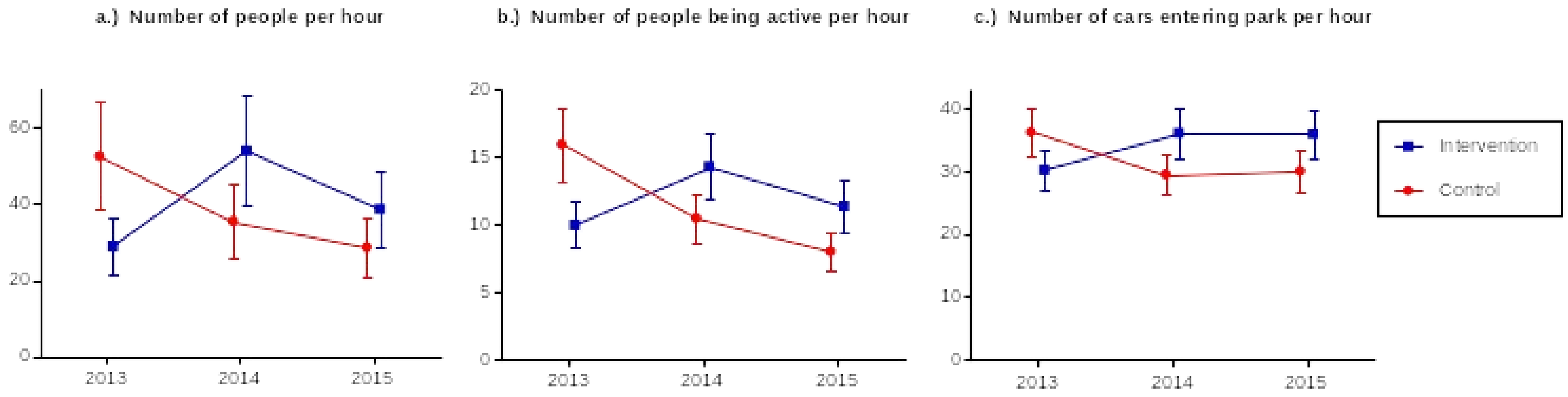
a.) Number of people per hour
0
20
40
60
2013
2014
2015
b.) Number of people being active per hour
0
5
10
15
20
2013
2014
2015
c.) Number of cars entering park per hour
0
10
20
30
40
2013
2014
2015
Intervention
Control
